# The Dose Makes the Poison: A Case Report of Acquired Methemoglobinemia

**DOI:** 10.3390/ijerph17061845

**Published:** 2020-03-12

**Authors:** Giulia Cannata, Luciana Abate, Chiara Scarabello, Monica Rubini, Alessandra Giacometti, Nicola Principi, Susanna Esposito, Icilio Dodi

**Affiliations:** 1 Pediatric Clinic, Pietro Barilla Children’s Hospital, Department of Medicine and Surgery, University of Parma, 43126 Parma, Italy; cannata.giulia@gmail.com (G.C.); luciana.abate@studenti.unipr.it (L.A.); cscarabello@ao.pr.it (C.S.); rubinim@ao.pr.it (M.R.); agiacometti@ao.pr.it (A.G.); idodi@ao.pr.it (I.D.); 2 Università degli Studi di Milano, 20122 Milan, Italy; nicola.principi@unimi.it

**Keywords:** cyanosis, CO-oximetry, hemogasanalysis, methemoglobin, methemoglobinemia, methylene blue

## Abstract

**Background:** Methemoglobinemia (MET) should be suspected in cases where cyanosis is not associated with signs and symptoms of lung and/or heart disease, or in a cyanotic child exhibiting discrepancies in the partial pressure of oxygen in the arterial blood, the blood oxygen saturation, and the clinical assessment. **Case presentation:** A 10-month-old girl was taken to the Pediatric Emergency Department for the acute, sudden development of significant peroral cyanosis associated with gray pigmentation of the skin. The problem was evidenced approximately one hour after she ingested a homemade puree of mixed vegetables, mainly composed of potatoes and chards that had been prepared three days before and had been kept in the refrigerator since then. Physical examination revealed that the child was very pale, conscious, and without respiratory distress. Oxygen saturation of hemoglobin in the arterial blood (SpO_2_) was 94%. Respiratory, cardiovascular, and abdominal evaluations did not reveal any signs of disease. A venous blood sample showed chocolate-colored blood with a pH of 7.404, a partial pressure of CO_2_ (pCO_2_) of 40.6 mmHg, a partial pressure of oxygen (pO_2_) of 21.3 mmHg, a bicarbonate level of 24 mmol/L, and an oxygen saturation (SO_2_%) of 47.7%. CO-oximetry carried out simultaneously identified a methemoglobin level of 22%. MET was suspected, and oxygen via nasal cannula at a rate of 4 L/min was given with only a slight increase in oxygen saturation (96%). Slow intravenous injection of methylene blue 1 mg/kg over a period of 5 min was initiated. The peripheral oxygen saturation (SpO_2_) gradually improved to 100% over the next 20 min. Forty minutes later, venous blood gas analysis showed a methemoglobin level of 0.9% with a complete resolution of cyanosis; supplemental oxygen via nasal cannula was therefore discontinued. During the next 36 h, the patient remained hemodynamically stable with good oxygenation on room air. **Conclusions:** This case report shows that recognition of acquired MET in a child with sudden cyanosis onset requires a high index of suspicion. In daily activities, there is a need to pay particular attention when homemade vegetable soups for child alimentation are prepared. The consumption of vegetable soups must occur immediately after preparation. Storage in a refrigerator must last no more than 24 h and if longer storage is needed, vegetable soups should be frozen.

## 1. Introduction

Cyanosis, i.e., a bluish discoloration of the skin and mucosa, results from the presence of at least 5 g/dL of deoxygenated hemoglobin in the circulation. This pathologic condition can be due to four different mechanisms: pulmonary venous desaturation, extrapulmonary right to left shunting, transposition physiology, and hemoglobin disorders affecting the affinity to oxygen [1]. Diagnostic differentiation among potential pathogenetic mechanisms is based on clinical history and a detailed clinical examination [2]. Hemoglobin disorders of oxygen affinity, mainly methemoglobinemia (MET), should be suspected in cases where cyanosis is not associated with signs and symptoms of lung and/or heart disease, or in a cyanotic child exhibiting discrepancies in the partial pressure of oxygen in the arterial blood, the blood oxygen saturation, and the clinical assessment.

MET is a rare blood disease in which the iron component of hemoglobin is oxidized from the ferrous to the ferric state, leading to a reduced release of oxygen in the tissues [3]. Clinical manifestations of MET increase with increasing methemoglobin levels. When these levels are between 10% and 20%, cyanosis can be appreciated. Values between 20% and 50% are associated with respiratory problems, dizziness, headache, and fatigue. Higher concentrations induce lethargy and can lead to death [3]. MET can be congenital or acquired. Two types of congenital METs have been identified, and both are chronic diseases. Type 1 is characterized by cyanosis since birth. In type 2, severe, progressive neurodevelopment alterations with death in the first years of life are the main clinical manifestations. This means that the sudden, unexpected appearance of cyanosis in a child without lung and heart disease strongly suggests acquired MET.

Several conditions are potential inducers of MET. Among them are acute diarrhea; consumption of high-nitrate water and food; use of certain drugs such as topical anesthetic agents, silver nitrate, sulfonamides, phenacetine, and sodium valproate; or exposure to aniline dyes, coloring compounds, or cleaning solutions [4,5,6,7,8]. Prompt identification of the etiology of acquired MET is essential to restore normal tissue oxygenation and metabolism, avoid long-term consequences, and reduce the risk of further episodes. Here, we report a case of acquired MET strictly related to vegetable consumption, from which a number of suggestions for infant food preparation can be derived.

## 2. Case Report

A 10-month-old girl was taken to the Pediatric Emergency Department of the Children’s Hospital of the University of Parma, Parma, Italy, for the acute, sudden development of significant peroral cyanosis associated with gray pigmentation of the skin. No other signs or symptoms of disease were reported by her parents. The problem was evidenced approximately one hour after she ingested a homemade puree of mixed vegetables, mainly composed of potatoes and chards that had been prepared three days before and had been kept in a refrigerator since then.

At admission, it was defined that both the family history and the personal history of the patient were negative for previous significant diseases. In particular, no recent episode of diarrhea was reported. Moreover, it was established that the family was living in an area with continuous control of potable water, the girl had not taken any kind of drugs, and there had been no accidental ingestion of foreign substances present in the house for personal hygiene and room cleaning. Physical examination revealed that the child was very pale, conscious, and without respiratory distress. Body temperature was 36.8 °C, heart rate was 140 beats/min, blood pressure was 90/60 mmHg, respiratory rate was 34 breaths/min, and oxygen saturation of hemoglobin in the arterial blood (SpO_2_) was 94%. Respiratory, cardiovascular, and abdominal evaluations did not reveal any signs of disease. The chest X-ray and the electrocardiogram were negative. A venous blood sample was withdrawn when the patient breathed room air and showed chocolate-colored blood with a pH of 7.404, a partial pressure of CO_2_ (pCO_2_) of 40.6 mmHg, a partial pressure of oxygen (pO_2_) of 21.3 mmHg, a bicarbonate level of 24 mmol/L, and an oxygen saturation (SO_2_%) of 47.7%. CO-oximetry carried out simultaneously identified a methemoglobin level of 22%; oxyhemoglobin of 36.9%; and carboxyhemoglobin of 0.6%. MET was suspected, and oxygen via nasal cannula at a rate of 4 L/min was given with only a slight increase in oxygen saturation (96%). Slow intravenous injection of methylene blue 1 mg/kg over a period of 5 min was initiated. After a transient decrease during methylene blue administration, peripheral oxygen saturation (SpO_2_) gradually improved to 100% over the next 20 min.

Forty minutes later, venous blood gas analysis showed a methemoglobin level of 0.9% with complete resolution of cyanosis; supplemental oxygen via the nasal cannula was therefore discontinued. Additional comprehensive metabolic panel blood tests were unremarkable except for a slight elevation of lactate dehydrogenase (295 U/L, normal values: <248 U/L) and creatine phosphokinase (334 U/L, normal values: 0–200 U/L).

The patient was subsequently admitted to the ward for close monitoring with continuous pulse oximetry and serial methemoglobin level checks. During the next 36 h, the patient remained hemodynamically stable with good oxygenation on room air; venous gas analysis taken on air at 9 and 30 h post-admission showed methemoglobin levels of 0.7% and 0.5%, respectively. The patient had a full recovery and was discharged home.

Table 1 summarizes venous gas analysis pre-treatment with methylene blue, 40 min post-treatment, and 9 h post-treatment.

The management of this patient was approved by the Ethics Committee of Emilia-Romagna Area Vasta Nord (PED-2020-03), and both parents provided written informed consent for the evaluation of the child. The Ethics Committee of Emilia-Romagna Area Vasta Nord approved the publication of this case, and both parents provided written informed consent for the publication of this manuscript.

## 3. Discussion

This case report shows that recognition of acquired MET in a child with sudden cyanosis onset requires a high index of suspicion and a series of clinical and laboratory tests to exclude the possibility that cyanosis can depend on cardiovascular and/or respiratory problems. Any cyanotic-appearing patient without demonstrable respiratory or cardiac disease whose SpO_2_ does not normalize with the administration of supplemental oxygen should raise the suspicion of MET. Additional diagnostic clues include chocolate-brown-colored blood, a normal partial pressure of oxygen (PO_2_) on arterial blood gas, and an oxygen saturation gap greater than 5% between the arterial blood gas analysis and pulse oximetry.

Once suspected, MET diagnosis is confirmed via multiple-wavelength CO-oximetry by direct measurement of methemoglobin levels in the blood [3]. However, particular attention must be paid to the MET pathogenesis to avoid the risk of recurrence. In this case, MET seemed to be strictly derived from the ingestion of vegetables, as the other potential causes of acquired MET, such as diarrhea, use of water containing a high-nitrate concentration, or the use of drugs or incidental ingestion of oxidizing agents, were very unlikely according to the details collected from the patient’s family and personal history. A homemade vegetable puree given to the young girl shortly before cyanosis onset is the only potential cause of this clinical manifestation. Vegetables contain nitrates that are, at least in part, converted into nitrites by gut microbiota. Nitrites are rapidly absorbed, and because they are potent oxidant agents, they cause methemoglobin formation. However, under physiological conditions, the amount of methemoglobin formed is promptly reduced through red cell nicotinamide adenine dinucleotide reductase activity, and approximately 99% of hemoglobin remains available to carry oxygen.

Risk of MET development occurs when the diet is mainly based on vegetables containing very large amounts of nitrates, and/or when the storage of previously prepared vegetable-based foods is suboptimal. Infants under 6 months of age are the subjects with the greatest risk, as they have low stomach acid production, more nitrate-reducing bacteria in the gut microbiota, incomplete development of the methemoglobin reductase system, and a certain amount of fetal hemoglobin that is more easily oxidized [9,10,11]. However, as weaning is not recommended before the 6th month of life [12], vegetable-induced MET may occur in older children in whom it is likely that some of the factors that favor nitrite formation tend to persist [13]. Among vegetables, leaf crops (e.g., spinach, lettuce, cabbage, and chards) have the highest mean nitrate levels, whereas potatoes, carrots, green beans, and zucchini generally have a lower nitrate content. However, it must be highlighted that MET can also occur with storage organs such as carrots [14], as the nitrate content can be higher than usual due to the season, soil composition, farming, and type and quantity of fertilizers used. Moreover, the final composition of homemade infant food can significantly change when storage is inappropriate, or when soup is consumed several hours after preparation. Contamination can occur, and a greater bacterial load can induce the production of large amounts of nitrites, favoring MET development. This has been found to be possible even when a vegetable puree is maintained in a refrigerator and consumption is delayed for more than 24 h [14].

## 4. Conclusions

Although the potential hazard of vegetable-borne nitrate for infants is already known, considering the increase in the number of infants and children who receive vegan or vegetarian diets [15], useful suggestions can be derived from this case report. First, there is a need to pay particular attention when homemade vegetable soups for child alimentation are prepared. The risk of MET is not limited to children under 6 months of age but also includes older infants, such as the one reported here. The content of leaves from leafy vegetables must be limited, and multiple administrations during the day must be avoided. It has been suggested that no more than 5–10 leaves of leafy vegetables such as chards and spinach should be included [16]. Moreover, the consumption of vegetable soups must occur immediately after preparation. Storage in a refrigerator must last no more than 24 h. Finally, if longer storage is needed, vegetable soups should be frozen.

## Figures and Tables

**Table 1:** Venous gas analysis pre-treatment with methylene blue, 40 min post-treatment, and 9 h post-treatment.

Venous Blood Gas	Pre-Treatment	40 min Post-Treatment	9 h Post-Treatment
pH	7.404	7.395	7.363
PCO_2_ mmHg	40.6	39.4	42.4
PO_2_ mmHg	21.3	28	29.5
ctHb g/dL	12	12	12.5
sO_2_ (%)	47.7	53.3	57.2
FO_2_Hb (%)	36.9	52.7	56,6
FCOHb (%)	0.6	0.2	0.4
FHHb (%)	40.5	46.2	42.3
FMetHb (%)	22	0.9	0.7
cLat mmol/L	1.8	1.3	1.3
cBase (Ecf),c mmol/L	0.7	-0.6	−1.1
cHCO_3_^−^ (P,st),c mmol/L	24	23	22.5
cK^+^ mmol/L	4.7	4.6	4.3
cNa^+^ mmol/L	138	139	141
cCa^2+^ mg/dL	5.51	5.55	5.44
cCl- mmol/L	106	107	108
